# Enhanced correlation-based linking of biosynthetic gene clusters to their metabolic products through chemical class matching

**DOI:** 10.1186/s40168-022-01444-3

**Published:** 2023-01-23

**Authors:** Joris J. R. Louwen, Marnix H. Medema, Justin J. J. van der Hooft

**Affiliations:** 1 Bioinformatics Group, Wageningen University & Research, 6708 PB Wageningen, the Netherlands; 2 Department of Biochemistry, University of Johannesburg, Johannesburg, 2006 South Africa

**Keywords:** Multi-omics, Genome mining, Genomics, Metabolome mining, Metabolomics, Chemical compound classification, Natural products, Specialised metabolites

## Abstract

**Background:**

It is well-known that the microbiome produces a myriad of specialised metabolites with diverse functions. To better characterise their structures and identify their producers in complex samples, integrative genome and metabolome mining is becoming increasingly popular. Metabologenomic co-occurrence-based correlation scoring methods facilitate the linking of metabolite mass fragmentation spectra (MS/MS) to their cognate biosynthetic gene clusters (BGCs) based on shared absence/presence patterns of metabolites and BGCs in paired omics datasets of multiple strains. Recently, these methods have been made more readily accessible through the NPLinker platform. However, co-occurrence-based approaches usually result in too many candidate links to manually validate. To address this issue, we introduce a generic feature-based correlation method that matches chemical compound classes between BGCs and MS/MS spectra.

**Results:**

To automatically reduce the long lists of potential BGC-MS/MS spectrum links, we match natural product (NP) ontologies previously independently developed for genomics and metabolomics and developed NPClassScore: an empirical class matching score that we also implemented in the NPLinker platform. By applying NPClassScore on three paired omics datasets totalling 189 bacterial strains, we show that the number of links is reduced by on average 63% as compared to using a co-occurrence-based strategy alone. We further demonstrate that 96% of experimentally validated links in these datasets are retained and prioritised when using NPClassScore.

**Conclusion:**

The matching genome-metabolome class ontologies provide a starting point for selecting plausible candidates for BGCs and MS/MS spectra based on matching chemical compound class ontologies. NPClassScore expedites genome/metabolome data integration, as relevant BGC-metabolite links are prioritised, and researchers are faced with substantially fewer proposed BGC-MS/MS links to manually inspect. We anticipate that our addition to the NPLinker platform will aid integrative omics mining workflows in discovering novel NPs and understanding complex metabolic interactions in the microbiome.

Video Abstract

**Supplementary Information:**

The online version contains supplementary material available at 10.1186/s40168-022-01444-3.

## Background

Complex microbial communities are nearly everywhere and rely on specialised metabolites to mediate host-microbe and microbe-microbe interactions. Such specialised metabolites, also called natural products (NPs), cover vast numbers of different scaffolds that constitute an incredible chemical diversity. Microbially derived NPs are also a prolific source for many types of drugs, such as antibiotics and anticancer agents [1]. This explains the drive to mine the microbiome for novel NPs and understand its largely unexplored and complex chemical interactions. Currently, the most common technique for analysing the microbial metabolome is liquid chromatography followed by tandem mass spectrometry (MS/MS or MS2), allowing for the discovery and structural annotation of NPs in complex mixtures [2, 3]. Especially in microbes, the biosynthetic pathways for synthesising NPs are often encoded by physically clustered sets of genes in the genome, known as biosynthetic gene clusters (BGCs). With the growing availability of genomic data in the last decade, multiple genome mining approaches for the identification of BGCs have appeared, such as antiSMASH and DeepBGC [4, 5].

Not only is the availability of metabolomic and genomic data growing independently, but also paired datasets of both types of omics data are currently recorded in platforms such as the Paired Omics Data Platform (PoDP) [6]. Leveraging genomic and metabolomic data together facilitates rapidly assessing the novelty of metabolites and linking them to their producing organisms and biosynthetic loci [7, 8]. Identifying candidate biosynthetic genes for a given metabolite provides complementary structural information inferred from the genome and metabolome for structural elucidation. While promising, such integrative omics mining is still challenging: despite community efforts like MIBiG [9], the typical number of validated paired data points, for which both the gene cluster and MS/MS spectral data of the metabolites produced from it are recorded, available within one experiment generally remains low. This hampers training and validation of integrative genome-metabolome mining strategies. One route to partially solve this challenge is by focusing on well-known and well-understood natural product classes [7]; however, this would severely decrease the discovery potential for novel chemistry. Therefore, generalised methodologies are required that can identify links between BGCs and metabolites without the necessity for large amounts of highly specific genetic or biochemical labelled data.

Recently, the NPLinker platform was developed for the systematic linking of MS/MS spectra and BGCs [10]. Currently, the standardised Metcalf score, a co-occurrence-based strain correlation score (i.e., using presence/absence patterns of strains that contain the BGC and/or the MS/MS spectrum), constitutes the main generic method to link BGCs and MS/MS spectra. To facilitate the linking process, NPLinker can currently integrate the output of molecular networking through Global Natural Products Social Molecular Networking (GNPS), which dereplicates and clusters MS/MS spectra into molecular families (MFs), with the output of BiG-SCAPE, which groups BGCs into gene cluster families (GCFs) [11, 12]. The MS/MS spectra or MFs and GCFs are used as inputs for two different scores: a co-occurrence-based score (originally devised by Doroghazi et al. [13]) that essentially considers if a GCF and a spectrum (or MF) occur together in the same strains, and a feature-based score that relies on (typically few) matches with public reference libraries. Unfortunately, the main co-occurrence-based score often produces a large list of potential links per GCF or spectrum, mainly because many BGCs are co-conserved in the same strains across long periods of evolutionary time [14]. This makes it hard to prioritise and find correct links, even when using reasonable cut-offs on the co-occurrence score. Hence, additional complementary scoring systems are needed to trim the list to a comprehensible length.

Apart from co-occurrence-based scoring, so-called feature-based scores that rely on annotated or inferred information of BGCs and MS/MS spectra could be used [7]. However, the current feature-based score that is implemented in NPLinker only solves the problem of long candidate lists in the few cases when there is sufficient similarity to known BGCs and spectra, as it compares BGCs and mass spectra to entries in MIBiG, a repository of experimentally validated BGCs [9, 15]; BGCs and mass spectra are then linked if they both have high similarity to (mass spectra associated with the product(s) of) the same MIBiG entry. Hence, it is often still challenging to find links when prioritising for novel chemistry, even within well-known NP classes such as non-ribosomal peptides (NRPs) or polyketides (PKs). Hence, in this work, we developed a novel concept for integrative omics mining that allows for generic chemical compound class matching to complement currently existing generic co-occurrence-based scores.

Instead of using similarity to entries in public reference libraries, we can obtain general knowledge about the structure of an unknown NP in the form of likely occurring scaffolds or substructures by using chemical compound classification strategies and using that to filter for (more) plausible candidates based on matching chemical compound classes. Over the last years, several general chemical compound classification ontologies have been constructed. One of these is ClassyFire: a hierarchical ontology consisting of superclass, class, and subclass categories and seven more detailed levels [16]. As an example, many PKs are classified at the superclass level as ‘phenylpropanoids and polyketides’, benzenoids, or lipids and lipid-like molecules. Recently, NPClassifier was developed specifically for NP classifications, taking both chemistry and biosynthetic pathways into account [17]. Compared to ClassyFire, it tailored the categorisation of NPs into seven major pathways, such as polyketides and ‘amino acids and peptides’, which are subsequently split into more detailed superclasses and classes, like macrolides and polyene macrolides. Furthermore, predicting these structure-based ontologies directly from MS/MS spectra has also become possible through CANOPUS and MolNetEnhancer [18, 19]. Similarly, chemical classifications can also be derived from genomic sequences, as antiSMASH uses class-specific detection rules to detect different types of BGCs, such as various types of PK synthases and NRP synthetases. However, a connection between structure- and genome-based classifications is currently still missing, as the ontologies from both classifications are not directly comparable.

**Fig. 1:**
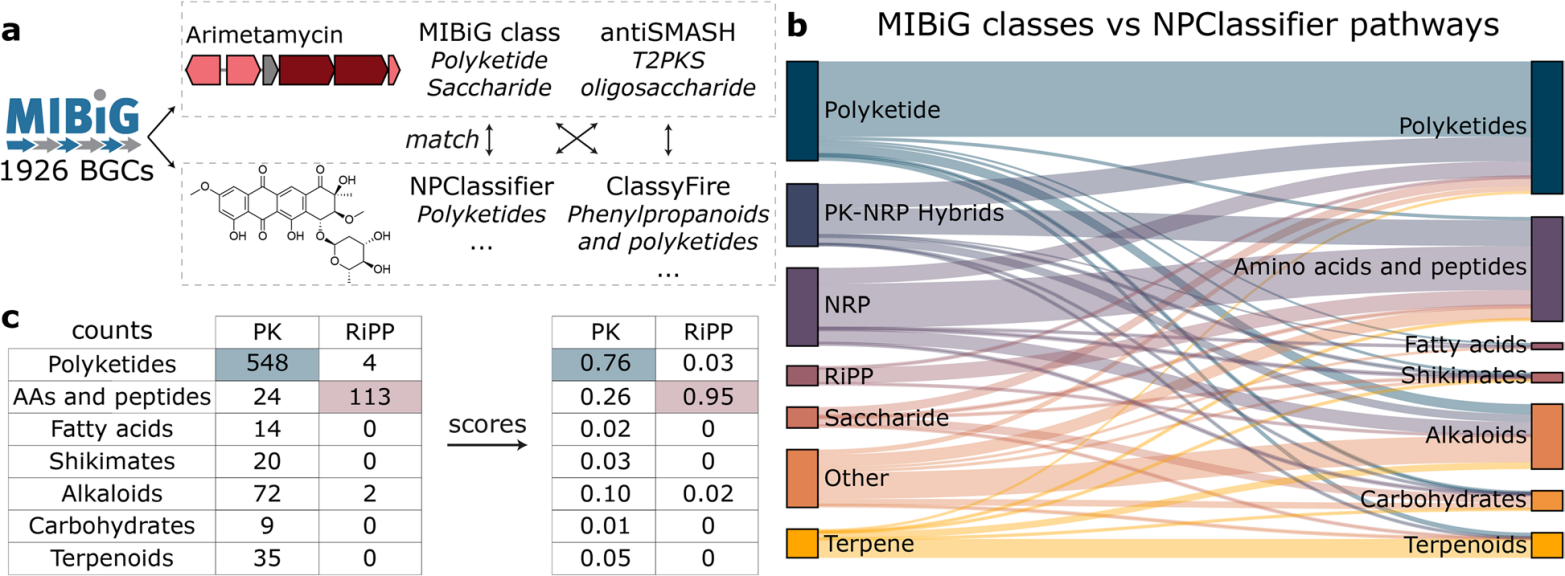
**a** 1926 MIBiG BGCs with corresponding structures were queried to antiSMASH, NPClassifier, and ClassyFire to retrieve BGC and chemical compound classifications. The arimetamycin MIBiG entry (BGC0000199) is given as an example with part of its BGC and the arimetamycin B chemical structure, for which the chemical compound classes are determined and the genome-based and structure-based classifications are matched. Please note that the NPClassifier and ClassyFire ontologies have more class terms than shown here. **b** Sankey diagram showing the relationships between matched MIBiG and NPClassifier pathway class terms. For reference, 722 BGCs had a Polyketide MIBiG class, 548 of which matched to NPClassifier pathway Polyketides. **c** Example scoring matrix between MIBiG classes and NPClassifier pathways, where each count is divided by the total occurrence of the class, in this case the column, to compute a score (see Eqs. 1 and 2 in the ‘Implementation’ section)

To reduce the number of false-positive BGC-MS/MS links in multi-omics analyses and thus accelerate NP discovery, we here introduce an automated approach to match these BGC and compound class ontologies. We used the matched class ontologies as a basis for the development of NPClassScore: NPLinker Class-based matching Score for linking BGCs and MS/MS-spectra. We also implemented NPClassScore in the NPLinker platform. This served two main purposes: (i) the impact of the novel score could be more easily addressed building on the existing NPLinker workflow and (ii) NPClassScore is also available for NPLinker users as a new generic feature-based linking score to remove unlikely BGC-MS/MS links predicted by correlation-based scores. To bridge the different class ontologies resulting from genome and metabolome mining, we used the MIBiG repository that contains experimentally validated BGCs and their products: antiSMASH genome-based classifications and manually annotated genome-based MIBiG classifications of the BGCs were matched to the chemical structure ontologies NPClassifier and ClassyFire [9] (Fig. 1a). The chemical compound class matching was automated by counting the genome-metabolome ontology connections between all the class terms for each MIBiG entry and using relative counts to assess the validity of matching class terms for the genome- and structure-based ontologies (Fig. 1b, c and Eqs. 1 and 2 in the ‘Implementation’ section). Here, using three use cases available from the Paired Omics Data Platform (PoDP), we demonstrate that these automatically matched ontologies are sensible as well as effective in removing irrelevant candidate links between BGCs and MS/MS spectra, while prioritising previously verified BGC-MS/MS spectra-metabolite links in three paired omics datasets from actinobacteria and cyanobacteria.

## Results and discussion

### Matching class ontologies between known BGC-structure pairs

Currently, there are 1926 experimentally validated BGCs with their corresponding structures present in the MIBiG v2.1 repository [9]. We used the manually annotated MIBiG classes and antiSMASH 5 class predictions for the BGCs alongside NPClassifier and ClassyFire class assignments for the structures to count all interactions between biosynthetic and structural classes (Fig. 1a). Based on the prevalence in MIBiG, we can then infer which class terms match frequently between the different ontologies. We note that the NPClassifier ontology is designed with natural products in mind, thus taking both chemistry and biosynthetic context into account, indeed leading to more direct matches between genome-based and structure-based classifications (Fig. 1b). For example, 76% of polyketide BGCs match to the NPClassifier ‘Polyketides’ pathway, most RiPPs and NRPs match to the ‘Amino acids and Peptides’ pathway, and 65% of terpene BGCs match to the ‘Terpenoids’ pathway. The most general superclass ontology of ClassyFire seems to be less suitable, as matches are more sparsely distributed across the different superclasses (Fig. S1). For instance, polyketide BGCs are distributed almost equally across five different ClassyFire superclasses. However, we also note that a certain degree of complementarity between the two chemical compound ontologies does exist, since, for example, NRPs match for 75% to the ‘Organic acids and derivatives’ superclass from ClassyFire, which is higher than for the NPClassifier pathway ontology.

At more detailed structure-based classifications levels, like the NPClassifier superclass or class levels, matches between genome-and structure-based classifications become more distributed as there are more options, and small distinctions within the classifications; hence, some structure-based ontologies are not reflected in current automated antiSMASH BGC classifications. For example, different type 1 polyketide synthase products, like products with the NPClassifier superclasses ‘Macrolides’, ‘Aromatic polyketides’, and ‘Linear polyketides’, now match to the generic type 1 polyketide synthase BGC class resulting in matches that are less conclusive (Fig. S2-S3). Another difficulty for class matching is the fact that many different hybrid classes exist that will make it impossible to reach perfect matches between most classes. Some NPs consist of very complex tailored scaffolds for which a combination of different types of biosynthetic machinery is needed, resulting in complex MIBiG classes like ‘Polyketide-NRP-Other’ for bromoalterochromide A. Additionally, some MIBiG records have very loose cluster boundaries with flanking genes that can trigger erroneous antiSMASH rules, and may therefore lead to erroneous biosynthetic class assignments. In contrast, the structure-based classifications are less affected by the presence of different structural scaffolds, as ClassyFire has a priority system to only consider the most important class terms and NPClassifier will only return at most two terms for its class level. However, the presence of multiple functional groups can sometimes cause challenges in putting a structure in ‘one’ chemical compound class. Nevertheless, both genome- and metabolome-based chemical compound classification systems seem to work well for most structures (Fig. 1). Furthermore, the more characterised BGCs will be deposited in MIBiG, the more these difficulties are expected to average out and improve the class matching usefulness. Similarly, depositing BGCs from a larger variety of classes will address biases in current data availability, as some classes, such as PKSs, are more abundant in the MIBiG database than others.

### NPClassScore can filter out many false-positive links

Based on the matched genome-based and structure-based chemical compound class ontologies, we constructed NPClassScore: the NPLinker Class-based matching Score for linking BGCs and MS/MS-spectra. We directly implemented NPClassScore in the NPLinker platform, where it can be used as an additional filtering step to assess the validity of a predicted link between a BGC or gene cluster family (GCF), and an MS/MS spectrum or molecular family (MF) [10]. NPClassScore consists of a scoring table for each of the 28 pairs of the matched genome- and structure-based ontology levels derived from MIBiG. The scores in the tables are made by dividing the counts for each class match by the total occurrence of either the genome-based class or the structure-based class (Fig. 1c; Table S1). This resulted in two different sets of scoring tables, one set that is used when starting from the genome side and one set that is used when starting from the metabolome side. NPClassScore takes the genome-based and structure-based classes from a proposed link as input, looks up the matching scores between these two classifications in the scoring tables, and reports the class match with the highest score from one of the scoring tables (Eqs. 1 and 2 in the ‘Implementation’ section). Thus, the NPClassScore indicates how plausible the link is between a BGC and a possible product based on how often their classes match among BGCs from MIBiG and their experimentally validated metabolic products.

**Fig. 2:**
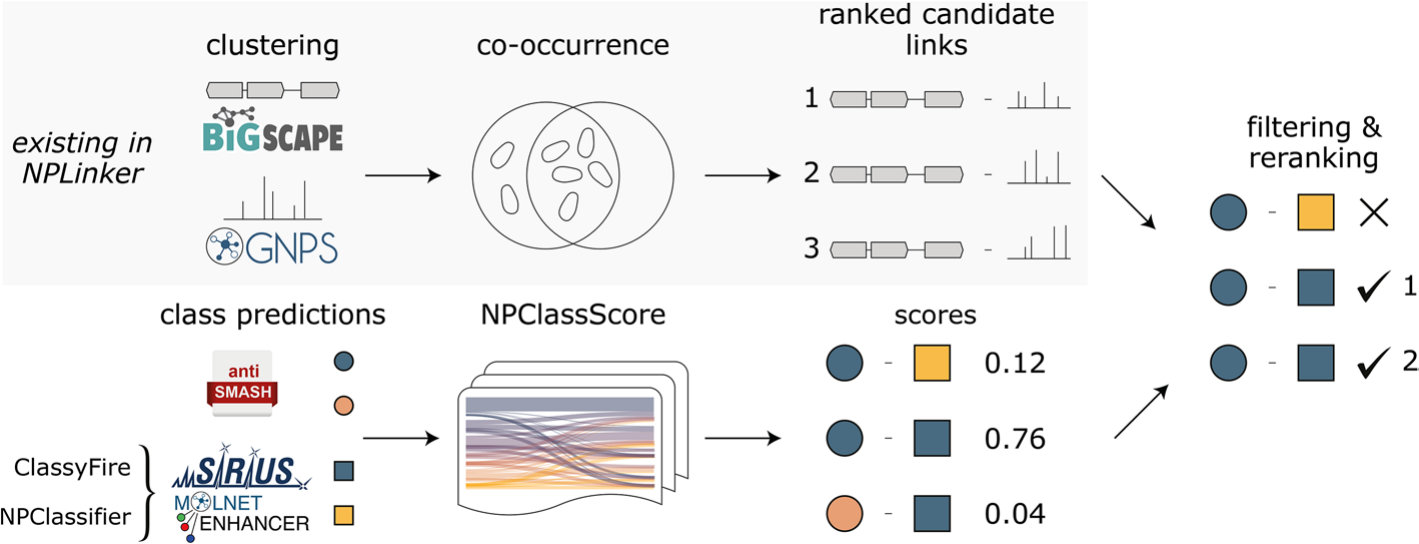
Schematic overview of the use of NPClassScore in integrative omics mining where functionality that previously existed in the NPLinker workflow is shaded in grey. First, BGCs and MS/MS spectra are clustered and dereplicated by BiG-SCAPE and GNPS molecular networking, respectively. Co-occurrence scoring (standardised Metcalf) is used to generate ranked candidate links of BGC-MS/MS spectra by correlating the presence/absence patterns of strains that contain a BGC and/or MS/MS spectrum. Depicted in the non-shaded area is the NPClassScore workflow which we integrated in the NPLinker platform. We incorporated structure-based classification predictions into the integrative omics mining workflow using CANOPUS from the SIRIUS platform and MolNetEnhancer, which predict ClassyFire and NPClassifier ontologies, while using antiSMASH and BiG-SCAPE for genome-based chemical compound classification ontologies. Based on the predicted classes of a BGC and MS/MS spectrum, NPClassScore outputs a score based on the matched genome- and structure-based ontologies in MIBiG. The best use of the scores from NPClassScore is to filter candidate BGC-MS/MS spectrum links based on a NPClassScore cut-off and then rerank the previously ranked candidate lists resulting from co-occurrence scoring

Predicted antiSMASH classes are directly used as input for NPClassScore and the general BiG-SCAPE classes are converted to MIBiG classes (Tables S2–S3). In order to predict ClassyFire and NPClassifier ontologies from MS/MS spectra, we used predictions from CANOPUS and MolNetEnhancer within NPClassScore (Fig. 2) [18, 19]. CANOPUS is a command-line tool that is part of the SIRIUS platform and can very accurately predict compound classifications if the right fragmentation trees are calculated. We implemented CANOPUS to run within NPLinker, but as it depends on calculating fragmentation trees, especially time-wise, it is only suitable to be used for the lower masses, below 850 Da. To also capture compound classifications for masses above 850 Da, we used MolNetEnhancer, which relies on propagating annotations between MS/MS spectra within MFs. Currently, MolNetEnhancer only provides ClassyFire predictions and has to be run on the GNPS platform, from which the results can be imported into NPLinker [12]. As the default for NPClassScore, MolNetEnhancer is used as well when there is no CANOPUS prediction for an MS/MS spectrum with a mass below 850 Da, but CANOPUS and MolNetEnhancer can also be used separately.

**Fig. 3:**
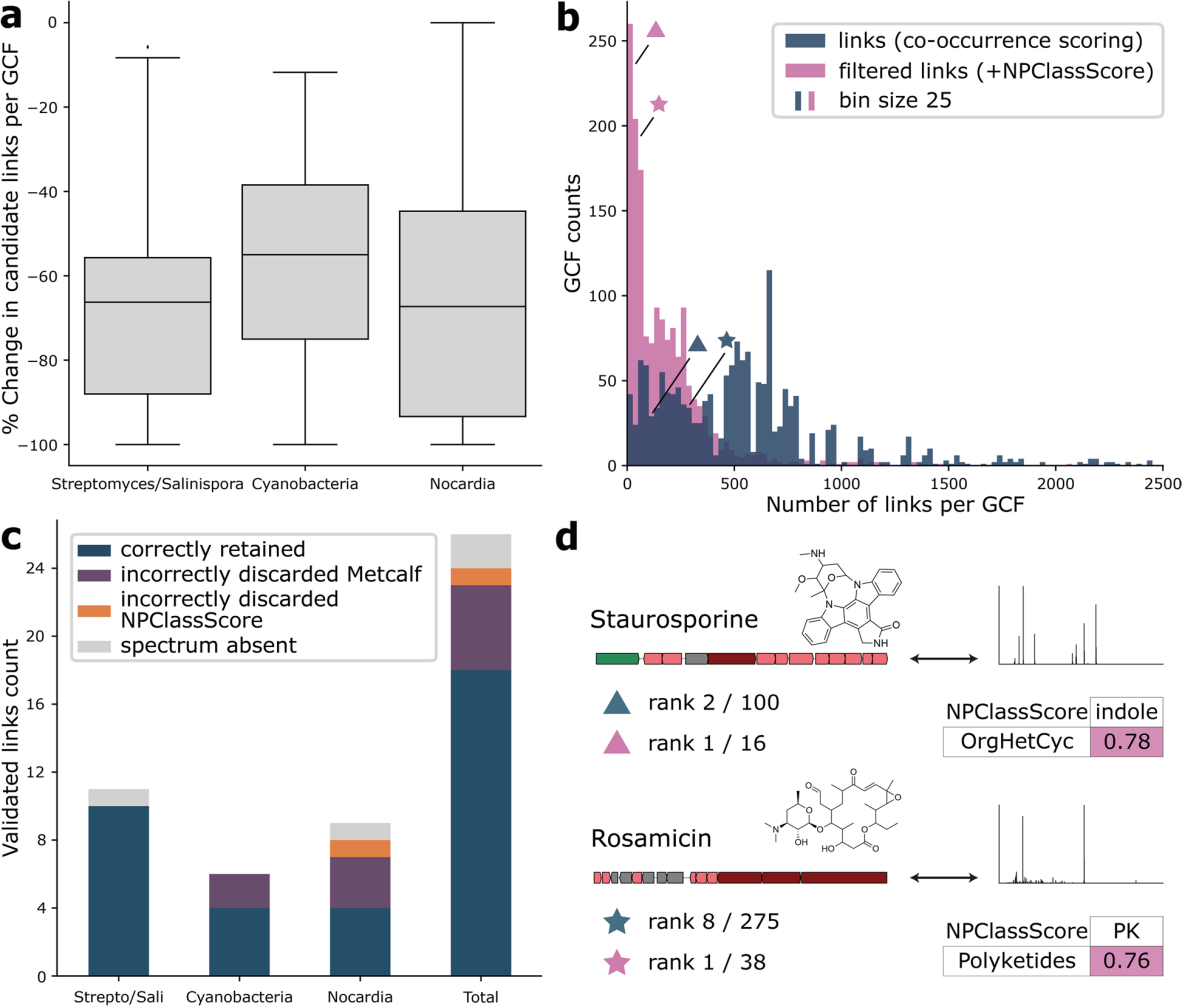
**a** The percentual decrease in the number of candidate links per GCF is shown for each dataset. Boxes are drawn from the first to the third quartile, separated by the median. Whiskers are extended to 1.5 times the interquartile range. **b** Histogram showing the number of candidate links per GCF in the Streptomyces/Salinispora dataset after co-occurrence scoring (standardised Metcalf), and after NPClassScore filtering with a cut-off of 0.25. The bin size is 25. The triangles and stars depict the number of links for the GCFs of staurosporine and rosamicin, respectively, as shown in **d**. **c** Summary for the detection of the experimentally validated links in the three datasets as listed on the PoDP. It is indicated whether links were correctly retained, incorrectly discarded due to a low standardised Metcalf score while passing the NPClassScore threshold of 0.25, or incorrectly discarded due to NPClassScore. Some validated links could not be detected as the reported spectrum on the PoDP was lacking in the dataset. Strepto/Sali is short for the Streptomyces/Salinispora dataset. **d** Depiction of two experimentally validated BGC-MS/MS links, for staurosporine and rosamicin, from the PoDP that are present in the dataset. The staurosporine-encoding BGC NC_009953.1.region013 from *Salinispora arenicola* CNS205 is shown as representative for GCF 534, linked to spectrum 89513. The rosamicin-encoding BGC NZ_AUGH01000019.region001 from *Salinispora pacifica* CNS237, which is fragmented due to being located on a contig edge, is shown as representative for GCF 944, linked to spectrum 130529. NPClassScore is depicted for both validated links as well as their ranks before and after filtering with NPClassScore, where OrgHetCyc is short for Organoheterocyclic compounds. Additionally, the total number of candidate MS/MS spectrum links are given for the staurosporine-and rosamicin-encoding GCF, denoted after the slash, before and after NPClassScore filtering. The number of links for the GCFs of staurosporine and rosamicin before and after NPClassScore filtering is also shown in **b** using the triangles and stars, respectively

To assess how well NPClassScore removes improbable links, we used NPLinker on three paired omics datasets from the PoDP that consist of 154 *Streptomyces* and *Salinispora* strains [20], 24 cyanobacterial strains [6], and 11 *Nocardia* strains [21] (Table S4). These are the largest datasets that contain multiple verified BGC-MS/MS-metabolite links in the PoDP, totalling 26 validated links across the three datasets. The three datasets will be referred to by their taxonomic descriptors indicated above. We analysed the three datasets separately using NPLinker and first used a co-occurrence-based strategy (standardised Metcalf score) to identify possible links between GCFs and MS/MS spectra, after which we used NPClassScore to filter the number of linked spectra per GCF [10, 13]. After filtering, the number of candidate MS/MS spectrum links for all GCFs decreased substantially for all datasets. In the Streptomyces/Salinispora dataset, the average number of candidate links per GCF decreased by 68% from 550 to 177. In the smaller Cyanobacteria and Nocardia datasets, the number of candidate links per GCF decreased from 27 to 13, and from 206 to 64, representing decreases of 53% and 69%, respectively. Averaging over the three datasets, this constitutes an average decrease in candidate links per GCF of 63% (Fig. 3a; Table S5). As the NPClassScore filtering depends on the chosen cut-off, we tried different cut-offs and decided on a cut-off of 0.25 as a default, as around this value there is a marked drop in the number of links per GCF for all datasets (Figs. S4-S6). This is also defendable from a theoretical perspective, as this cut-off means that a given class match should occur for at least 25% of the total occurrences of the class among MIBiG entries. Additionally, this threshold results in many more GCFs with manageable numbers of candidate MS/MS links, which can be analysed manually: in the large Streptomyces/Salinispora dataset, it yields 92 GCFs with 10 or fewer candidate links and 270 GCFs with 25 or fewer candidate links. In contrast, without filtering based on NPClassScore, only 5 GCFs would have fewer than 10 candidate links and only 42 GCFs would have fewer than 25 candidate links in the same dataset (Fig. 3b). Similar trends can be seen for the other two datasets (Fig. S7). Thus, using NPClassScore constitutes a real advantage for end-users as they can now realistically inspect a much larger percentage of predicted candidate links that are also more likely to be real.

One of the main reasons that the co-occurrence-based strain correlation score produced a substantial number of false positives is that it does not consider the type or function of the natural product. Feature-based scores such as NPClassScore are mostly complementary to strain-correlation-based scores, as they link BGCs and mass spectra based on different principles. Using feature-based scores alone would also yield many false positives, as then the actual co-occurrence across strains would not be considered at all. The effect of using just a co-occurrence-based strain correlation score (standardised MetCalf) as compared to using the combined co-occurrence and feature-based scores is demonstrated both in Fig. 3 and in Table 1.

**Table 1:** All validated links from the three datasets as listed on the PoDP. The standardised Metcalf score and NPClassScore of all the links are stated as well as the rank of the verified link in the candidate list before and after NPClassScore filtering. The rank number may be shared with a number of other links due to their scores being the same which is indicated in parentheses. Retimycin and nocardimicin have no information as their MS/MS spectra could not be located in their respective datasets

Name	Dataset	Rank NPClassScore (shared with n other links)	Rank Metcalf (shared with n other links)	Standardised Metcalf	NPClassScore
Staurosporine	Streptomyces/Salinispora	1	2	9.0	0.78
Rosamicin	Streptomyces/Salinispora	1 (6)	8 (38)	8.7	0.76
Desferrioxamine	Streptomyces/Salinispora	1	1	9.5	0.36
Rifamycin	Streptomyces/Salinispora	152	257	4.4	0.45
Lomaiviticin	Streptomyces/Salinispora	30 (18)	381 (151)	3.0	0.96
Arenimycin	Streptomyces/Salinispora	1 (4)	1 (12)	12.4	0.96
Enterocin	Streptomyces/Salinispora	1 (8)	4 (81)	12.4	0.40
Salinamide	Streptomyces/Salinispora	1 (40)	1 (84)	12.4	0.64
Cyclomarin	Streptomyces/Salinispora	3 (49)	4 (83)	8.7	0.91
Retimycin	Streptomyces/Salinispora	-	-	-	-
Actinomycin	Streptomyces/Salinispora	1 (97)	1 (159)	12.4	0.76
Anabaenopeptin	Cyanobacteria	2 (3)	3 (6)	4.2	0.93
Micropeptin	Cyanobacteria	-	Incorrectly discarded	-	1.00
Kawaguchipeptin	Cyanobacteria	1 (37)	2 (39)	4.7	0.71
Microcyclamide	Cyanobacteria	1 (13)	1 (18)	4.7	1.00
Microcystin	Cyanobacteria	11 (4)	13 (8)	3.2	0.64
Microcystin RR	Cyanobacteria	-	Incorrectly discarded	-	0.64
Mycobactin	Nocardia	1 (90)	2 (133)	3.2	0.64
Mycobactin	Nocardia	Incorrectly discarded	-	-	0.03
Nocardimicin	Nocardia	-	-	-	-
Nocobactin	Nocardia	-	Incorrectly discarded	-	0.59
Nocobactin	Nocardia	-	Incorrectly discarded	-	0.59
Nocobactin	Nocardia	-	Incorrectly discarded	-	0.64
Formobactin	Nocardia	1 (338)	4 (648)	2.12	0.64
Nocardimicin	Nocardia	2 (255)	11 (427)	2.12	0.59
Carboxynocobactin	Nocardia	1 (338)	4 (648)	2.12	0.46

### Validated links are retained and get higher ranks after NPClassScore filtering

To assess if the filtered links based on NPClassScore are sensible, we used our three selected paired omics datasets from the PoDP, in each of which several previously experimentally validated BGC-MS/MS spectral-chemical structure links had been recorded. In the three datasets, we checked whether these validated links were retained and whether they gained a higher rank in the lists of predicted links upon NPClassScore application. The Streptomyces/Salinispora, Cyanobacteria, and Nocardia datasets have 11, 6, and 9 validated links recorded on the PoDP. Out of the 26 total validated links, 2 could not be found due to a missing spectrum in the datasets. Of the remaining links, 23 out of 24 passed the default NPClassScore threshold, constituting an accuracy of 96% (Fig. 3c). Additionally, this confirms the choice for our default NPClassScore threshold of 0.25 as substantial numbers of validated links are removed beyond this threshold (Fig S8). As an example, from the Streptomyces/Salinispora dataset, we found a link between GCF 534 (present in 54 strains), and spectrum 89513 (present in 67 strains), representing the link for staurosporine based on the PoDP [6] (Fig 3d). With a co-occurrence score of 9.0, this link was initially ranked second in the list of 100 potential links for GCF 534. After filtering using NPClassScore, 16 potential links were left, and spectrum 89513 was ranked first; its NPClassScore was 0.78 from the antiSMASH-ClassyFire-superclass scoring table, matching indole to Organoheterocyclic compounds. Similarly, our analysis retrieved the validated link for rosamicin: the rosamicin-biosynthesis-associated GCF 944 (present in 2 strains) was linked to spectra 130529 and 141312, each of which was present in 1 of the 2 strains (Fig. 3d). With a co-occurrence score of 8.7, the links with both spectra were ranked at a shared eighth position in the list of 275 candidate links. After filtering using NPClassScore, 38 candidate links were left in total, and both spectra were jointly ranked at the first position; their NPClassScore scores were 0.76 from the MIBiG-NPClassifier-pathway scoring table, matching Polyketide to Polyketides.

Of note, 5 out of 26 validated links did not pass the co-occurrence scoring threshold implemented in NPLinker, meaning an accuracy of 75% for the entire NPLinker workflow including NPClassScore. Most probably, for these links, the clustering of MS/MS spectra and BGCs into dereplicated MS/MS spectra and GCFs do not agree with each other, i.e., their MS/MS spectra are similar enough to be clustered together but BGCs are not, or vice versa. An example that supports this hypothesis is nocobactin in the Nocardia dataset, where the actual link passed the NPClassScore cut-off with a score of 0.59, but the BGCs did not cluster together with our currently applied BiG-SCAPE cut-off, whereas the MS/MS spectra did cluster with the current parameter and threshold settings. Regarding the 21 correctly retained validated links, they were not only retained, but they also ranked higher in the lists with candidate links due to removing false-positive links (Table 1). Out of 21 validated links, 12 are even ranked at the first position after NPClassScore filtering, compared to 5 links being ranked first when just using co-occurrence-based scoring. This shows that after NPClassScore filtering, the candidate links that are retained at high rankings are more reliable and worth exploring manually.

It is good to note that by using multiple classification ontologies, and their multiple hierarchical levels at the same time, most BGCs and MS/MS spectra will have at least one match in the NPClassScore tables. This ensures that NPClassScore can almost always be used to assess the validity of a proposed link based on chemical class information. As such, NPClassScore is used as a lenient and generic filtering mechanism, as a potential link is already retained when there is a match in only one of the scoring tables that passes the threshold (coming from either the genome or metabolome). We do note that CANOPUS and MolNetEnhancer both give quite different chemical class predictions for the spectra in our dataset (Tables S6–S7). Looking at the 6606 spectra with predictions from both tools in the Streptomyces/Salinispora dataset, the ClassyFire superclass ‘Lipids and lipid-like molecules’ is predicted 2686 times by MolNetEnhancer and 1,301 times by CANOPUS. Similar stark differences can be seen for other superclasses like, ‘Organic acids and derivatives’ and ‘Phenylpropanoids and polyketides’. Although we show that NPClassScore can filter down the results and prioritise actual BGC-MS/MS spectrum links, the choice of software tool for structure-based ontology prediction will have a large influence on the final outcome.

## Conclusion

We made a substantial step forward in generic integrative genome-metabolome mining by enabling automated matching of genome-derived and structure-based chemical compound class ontologies and implementing the empirical NPClassScore to assess possible BGC-MS/MS spectrum links. To facilitate its use, NPClassScore is implemented in the NPLinker platform, and the NPClassScore scoring tables can be further updated upon new releases of MIBiG. For now, we rely mostly on CANOPUS as a predictive software for chemical classes from MS/MS spectra with lower masses, and MolNetEnhancer for larger-sized molecules. In the future, other methods, like mass-spectral embeddings such as Spec2Vec and MS2DeepScore, might be better suited to deal with bigger metabolites and decrease run times [8, 22, 23]. Although we made good efforts to show that most validated links are retained by NPClassScore in three datasets that contain many of the validated links in the PoDP, the lack of verified genome-metabolome links remains one of the current bottlenecks to validate new integrative omics mining methods. We anticipate that our method will help to record more validated links to move this field forward. Integrative genome-metabolome mining is a complex problem that will require many different solutions and smart ways to integrate those. Combining and streamlining NPLinker with other novel linking methods, such as NPOmix, will be key for advancing this field to understand complex microbial communities and prioritise NP discovery [24]. Other possible routes are to implement additional feature-based scores such as a score based on shared substructures as inferred to be present from the genomic and metabolomic data, the first for example through iPRESTO that finds sub-clusters in BGCs that likely encode for biosynthetic scaffolds or substructures [25], and the latter for example through the use of data-driven approaches that find (MS2LDA) or contain (MotifDB) mass spectral patterns that can be connected to chemical substructures [26, 27]. The class matching matrices developed here could also be used to select a reduced list of plausible candidates in structure databases for predicted BGCs based on matching classes. Our contribution will aid researchers in finding correct BGC-MS/MS spectrum links more easily and we think that NPClassScore will facilitate the acceleration of efforts to connect metabolites to their producer strains and elucidate their roles in microbial ecosystems and the microbiome.

## Implementation

### Matching class ontologies in MIBiG repository

All entries from MIBiG 2.0 were downloaded from mibig.secondarymetabolites.org in json format. The SMILES and MIBiG (sub)classes were extracted from the json files, and the predicted antiSMASH 5 classes for the MIBiG BGCs were retrieved from the MIBiG website. For each MIBiG entry, the ClassyFire and NPClassifier chemical compound classifications were retrieved through the GNPS API (ccms-ucsd.github.io/GNPSDocumentation/api/) using the SMILES or RDKit generated InChIKeys. ClassyFire classes were retrieved until the subclass level. Genome- and structure-based classes were related to each other by splitting up hybrid classes and counting the connections between all class terms, except for PKS-NRP hybrids that were grouped together at the MIBiG class level. The count of a pair of class terms from the genome-and structure-derived ontologies is then used to express the match between the pair of class terms. As different classes have different overall occurrences, we used the relative counts as a score to assess the validity for a match between a genome- and structure-based class. For each matching pair of class terms, the score was calculated by dividing the count of the match by the total occurrence of the class term, starting either from the genome-based class (Eq. 1) or from the structure-based class (Eq. 2).

1 $${\displaystyle \begin{array}{c}b=\mathrm{class}\ \mathrm{term}\ \mathrm{from}\ \mathrm{a}\ \mathrm{genome}\ \mathrm{based}\ \mathrm{ontology},\\ {}m=\mathrm{class}\ \mathrm{term}\ \mathrm{from}\ \mathrm{a}\ \mathrm{structure}\ \mathrm{based}\ \mathrm{ontology},\\ {}B=\left\{{b}_1\dots {b}_n\right\}:\mathrm{set}\ \mathrm{of}\ \mathrm{predicted}\ \mathrm{classes}\ \mathrm{for}\ \mathrm{a}\ \mathrm{BGC},\\ {}M=\left\{{m}_1\dots {m}_n\right\}:\mathrm{set}\ \mathrm{of}\ \mathrm{predicted}\ \mathrm{classes}\ \mathrm{for}\ \mathrm{an}\ \mathrm{MS/MS}\ \mathrm{spectrum},\\ {}{N}_b=\mathrm{number}\ \mathrm{of}\ \mathrm{MIBiG}\ \mathrm{BGCs}\ \mathrm{with}\ \mathrm{b},\\ {}{N}_{bm}=\mathrm{number}\ \mathrm{of}\ \mathrm{MIBiG}\ \mathrm{BGCs}\ \mathrm{with}\ \mathrm{b}\ \mathrm{that}\ \mathrm{encode}\ \mathrm{a}\ \mathrm{structure}\ \mathrm{with}\ \mathrm{m},\\ {}{\mathrm{NPClassScore}}_{\mathrm{BGC}\to \mathrm{MS}/\mathrm{MS}}\ \left(B,M\right)=\mathit{\max}\left\{\frac{N_{bm}}{N_b}\right\}:{b}_1\dots {b}_n,{m}_1\dots {m}_n\ \end{array}}$$

Equation 1: NPClassScore methodology for calculating the score between a BGC (or GCF) and an MS/MS spectrum (or molecular family) starting at the genome side. Let *b* be a predicted genome-based class term of a BGC and *B* the set of all *b* for a BGC. Let *m* be a predicted structure-based class term of an MS/MS spectrum and *M* the set of all *m* for an MS/MS spectrum. The match between a given *b* and *m* is expressed by the relative frequency of the number of MIBiG BGCs with *b* that encode a structure with *m*, given by *N*_*bm*_ divided by *N*_*b*_. The relative frequencies are stored in the scoring tables used by NPClassScore. The final score returned by NPClassScore is the maximum of all relative frequencies that arise between all combinations of values between *B* and *M*.

2 $${\displaystyle \begin{array}{c}{N}_m=\mathrm{number}\ \mathrm{of}\ \mathrm{MIBiG}\ \mathrm{structures}\ \mathrm{with}\ \mathrm{m},\\ {}{N}_{mb}=\mathrm{number}\ \mathrm{of}\ \mathrm{MIBiG}\ \mathrm{structures}\ \mathrm{with}\ \mathrm{m}\ \mathrm{that}\ \mathrm{are}\ \mathrm{encoded}\ \mathrm{by}\ \mathrm{a}\ \mathrm{BGC}\ \mathrm{with}\ \mathrm{b},\\ {}{\mathrm{NPClassScore}}_{\mathrm{MS/MS}\to \mathrm{BGC}}\ \left(M,B\right)=\mathit{\max}\left\{\frac{N_{mb}}{m}\right\}:{b}_1\dots {b}_n,{m}_1\dots {m}_n\ \end{array}}$$

Equation 2: NPClassScore methodology for calculating the score between an MS/MS spectrum (or molecular family) and a BGC (or GCF) starting at the metabolome side. Using the same definitions as in Eq. 1, the match between a given *m* and *b* is expressed by the relative frequency of the number of MIBiG structures with m that are encoded by a BGC with *b*, given by *N*_*mb*_ divided by *N*_*m*_. The relative frequencies are stored in the scoring tables used by NPClassScore. The final score returned by NPClassScore is the maximum of all relative frequencies that arise between all combinations of values between *M* and *B*.

From the 3 genome-based and 9 structure-based class levels, this created in total 54 scoring tables of two types, one set to use when coming from the genome side and one set to use when coming from the metabolome side, akin to NPLinker that can create a link between a BGC and spectrum or vice versa. Code and scoring tables are available at github.com/louwenjjr/mibig_classifications.

### Predicting chemical compound classifications from mass spectra

Currently, CANOPUS and MolNetEnhancer are used within NPLinker to predict ClassyFire and NPClassifier ontologies directly from MS/MS spectra. By default, CANOPUS is used for mass values below 850 Da, and MolNetEnhancer for mass values above 850 Da and for spectra without a CANOPUS prediction. CANOPUS and MolNetEnhancer can also be used separately. Sirius v4.9.3 is used to run CANOPUS directly on the mgf file resulting from GNPS molecular networking. By default, the ‘formula zodiac structure canopus’ setting and the ‘--maxmz’ cut-off set to 850 are used. The CANOPUS results are related to the spectra and molecular families (MFs) with classifications_to_gnps.py from canopus_treemap (github.com/kaibioinfo/canopus_treemap). For the CANOPUS predictions, the ClassyFire cut-off is 0.5 and the NPClassifier cut-off 0.33. All resulting ClassyFire classes are sorted by priority at each class level, while NPClassifier classes are sorted by their probabilities. To obtain classes for MFs, classes of all spectra belonging to an MF are counted at each level, and classes that occur in at least 20% of the spectra in the MF are kept. ClassyFire classes are again sorted by priority and NPClassifier classes by class occurrence in the MF. Currently, MolNetEnhancer has to be run externally, for example on the GNPS platform [12]. The output file ClassyFireResults_Network.txt can be downloaded from the GNPS platform and used directly as input for NPLinker and NPClassScore.

### NPClassScore in NPLinker

The MIBiG scoring tables are used in NPLinker v1.2 (github.com/NPLinker/nplinker) by the new scoring method NPClassScore, which can create links between BGCs or GCFs and MS/MS spectra or MF. Out of the 54 total scoring tables, 28 are used by NPClassScore, as some classes could not be predicted from BGCs or MS/MS spectra, like MIBiG-subclass and the is_glycoside class level from NPClassifier. Within NPClassScore, GCFs are annotated with an MIBiG class, based on their BiG-SCAPE class, and with antiSMASH classes of the children BGCs if the class occurs in at least half of the BGCs in the GCF (Tables S2–S3). Spectra and MFs get their ClassyFire and NPClassifier annotations from CANOPUS or MolNetEnhancer predictions. The scores are looked up in the scoring tables for all the different combinations of class ontologies and then returned from high to low, where the highest score is returned by NPClassScore and can be used to filter out candidate links after using other scoring methods such as co-occurrence-based scoring (Fig. 2). A demo notebook to perform NPClassScore linking within NPLinker is available at https://github.com/NPLinker/nplinker/blob/master/notebooks/npclassscore_linking/NPClassScore_demo.ipynb.

### Preparing the datasets and running NPLinker

The molecular network for the Streptomyces/Salinispora dataset used in Crüsemann et al. was cloned to GNPS version 28.2, which consists of MassIVE accessions: MSV000078836, MSV000078839, and MSV000079284, containing data for 159 strains of *Streptomyces* and *Salinispora* (gnps.ucsd.edu/ProteoSAFe/status.jsp?task=9ba6f1296adb494db4dac117110a420a) [20]. From these accessions in the Paired-omics Data Platform, the corresponding genomes were downloaded from NCBI if they had RefSeq or GenBank identifiers. On the 104 retrieved genomes, antiSMASH 6 was run and the output was merged with antiSMASH 3 data from the other 50 strains used in Crüsemann et al. [20]. The used strain mappings file was combined from the three PoDP accessions (github.com/NPLinker/nplinker/tree/master/notebooks/npclassscore_linking/crusemann_strain_mappings.csv). The Cyanobacteria and Nocardia datasets were accessed through their PoDP accessions, MSV000084950 and MSV000084771, respectively. The molecular networks listed in the PoDP were used, while antiSMASH 6 was run on the listed 24 and 11 genomes, respectively. CANOPUS was run for the three datasets within NPLinker with the aforementioned default settings, which took around 24 h for the Streptomyces/Salinispora dataset and less for the other two datasets. MolNetEnhancer was run for the three datasets on GNPS (https://gnps.ucsd.edu/ProteoSAFe/status.jsp?task=6a07ff87c7574329b397a779a716fc69, https://gnps.ucsd.edu/ProteoSAFe/status.jsp?task=bb106ef439094975a5035b5d7c7f762e, https://gnps.ucsd.edu/ProteoSAFe/status.jsp?task=1de521d0932f414682ef4052496ff8a6). NPLinker v1.2 was first run through the docker version with a BiG-SCAPE cut-off of 0.3, after which the datasets were further analysed within jupyter notebooks (https://github.com/NPLinker/nplinker/tree/master/notebooks/npclassscore_linking). Standardised Metcalf scoring was used with a cut-off of 2.5, after which candidate links were filtered out if their NPClassScore score was below a cut-off of 0.25. By using CANOPUS and MolNetEnhancer together, most MS/MS spectra were annotated with structure-based ontologies. We note that we discarded candidate links without structure-based classes, due to the 1988 MS/MS spectra across the datasets without a CANOPUS or MolNetEnhancer prediction (Table S4). We decided to do this as it did not hamper our effort of finding the validated links in the three datasets. Not filtering out such links results in an average decrease of 49% of the candidate links per GCF (Table S8). This functionality can be easily switched on or off by toggling the .filter_missing_scores attribute of the NPClassScore scoring method within NPLinker.

### Validating experimentally validated BGC-MS/MS spectrum links

To locate the BGCs for the validated links as listed in the PoDP, we used cblaster with default settings with the MIBiG BGC listed on the PoDP as query and the antiSMASH gbk files of each of the three datasets as database [28]. To identify the correct MS/MS spectra for the validated links, we found the MF, cluster, or scan id as listed on the PoDP in our molecular networks. If that failed, for example in the case where we created a new molecular network for the Streptomyces/Salinispora dataset, we compared parent masses that occurred in the same strains as listed on the PoDP. No MS/MS spectrum could be found this way for retimycin and nocardimicin. The ranked position for each validated link was recorded before and after filtering with NPClassScore, taking into account that ranks can be shared as multiple candidate links can have the same scores. We note that the Nocardia dataset exhibited quite low standardised Metcalf scores for the validated links, which is why we changed the standardised Metcalf threshold from 2.5 to 2 for this dataset to include the validated links. This is probably due to the Nocardia dataset being the smallest out of the three datasets, as well as due to the incongruence between the BGC and MS/MS spectrum clustering cut-offs.

## Availability and requirements

Project name: NPClassScore implemented in NPLinker.

Project home page: https://github.com/NPLinker/nplinker

Operating system(s): Platform independent

Programming language: Python

Other requirements: See requirements.txt at https://github.com/NPLinker/nplinker.

License: Apache-2.0 License.

Any restrictions to use by non-academics: See license.

## Supplementary Information

**Additional file 1: Figure S1.** MIBiG classes matched to ClassyFire superclasses. **Figure S2.** antiSMASH-predicted classes matched to NPClassifier superclasses, where matches with counts above five are shown. **Figure S3.** antiSMASH-predicted classes matched to ClassyFire classes, where matches with counts above five are shown. **Figure S4.** Number of links with MS/MS spectra per GCF for all the GCFs in the Streptomyces/Salinispora dataset after using standardised Metcalf scoring in combination with NPClassScore filtering at varying cut-offs for the NPClassScore. The standardised Metcalf score cut-off was 2.5. **Figure S5.** Number of links with MS/MS spectra per GCF for all the GCFs in the Cyanobacteria dataset after using standardised Metcalf scoring in combination with NPClassScore filtering at varying cut-offs for the NPClassScore. The standardised Metcalf score cut-off was 2.5. **Figure S6.** Number of links with MS/MS spectra per GCF for all the GCFs in the Nocardia dataset after using standardised Metcalf scoring in combination with NPClassScore filtering at varying cut-offs for the NPClassScore. The standardised Metcalf score cut-off was 2.5. **Figure S7.** Histograms showing the number of candidate MS/MS spectrum links per GCF in the (a) Cyanobacteria dataset and (b) Nocardia dataset after co-occurrence scoring (standardised Metcalf), and after NPClassScore filtering with a cut-off of 0.25. The bin sizes are 5 in (a) and 25 in (b). The results highlight how NPClassScore narrows down the number of candidate links for most GCFs. **Figure S8.** Number of retained validated MS/MS spectrum links versus the percentage of filtered out candidate links per GCFs with different NPClassScore cut-offs. The percentage of filtered out candidate links is an average over the three datasets. **Table S1.** Scoring table from NPClassScore showing the scores from MIBiG classes to NPClassifier pathways. **Table S2.** Translation of BiG-SCAPE to MIBiG classes. **Table S3.** Translation of antiSMASH classes from all other versions to match antiSMASH v5 classes. **Table S4.** Information about the contents of the three datasets. We included the number of spectra in our versions of the datasets separately as some spectra present in the molecular networks occurred in, for example, control samples, or samples we did not use. **Table S5.** The average number of MS/MS spectrum links per GCF for each dataset after NPClassScore filtering with different cut-offs, along with the percentual decrease in the number of links. In this case, the MS/MS spectra without structure-based predictions are automatically excluded. **Table S6.** Counts for the number of spectra with certain ClassyFire superclasses as predicted by MolNetEnhancer (MNE), MNE for spectra below 850 Da, and CANOPUS. Counts are coloured from white to red, white being the lowest count and red being the highest count for each column. **Table S7.** Counts of the ClassyFire superclass predictions for the 6,606 spectra that could be predicted by both MolNetEnhancer (MNE), and CANOPUS, showing the MNE predictions and CANOPUS predictions. Counts are coloured from white to red, white being the lowest count and red being the highest count for each column. **Table S8.** The average number of candidate links per GCF for each dataset after NPClassScore filtering with different cut-offs, along with the percentual decrease in the number of links. In this case, the MS/MS spectra without structure-based predictions are automatically included.

## Data Availability

All code used during this study is available on github at https://github.com/NPLinker/nplinker and https://github.com/louwenjjr/mibig_classifications. A demo notebook to perform NPClassScore linking within NPLinker and notebooks to replicate this study are available at https://github.com/NPLinker/nplinker/notebooks/npclassscore_linking. All data used in this study is public. The MIBiG json files analysed during the current study are available in the MIBiG repository, https://dl.secondarymetabolites.org/mibig/mibig_json_2.0.tar.gz. The molecular networking results analysed during the current study are available in the GNPS platform, https://gnps.ucsd.edu/ProteoSAFe/status.jsp?task=9ba6f1296adb494db4dac117110a420a, http://gnps.ucsd.edu/ProteoSAFe/status.jsp?task=cefe9408d6e64d7691490c7ac796fbea, and http://gnps.ucsd.edu/ProteoSAFe/status.jsp?task=a50dc9e2fc4f4a26aef72b0e05fdb123, for the Salinispora/Streptomyces, Cyanobacteria, and Nocardia datasets, respectively. The RefSeq and GenBank accessions for the *Salinispora* and *Streptomyces* strains analysed during the current study are available in the following PoDP accessions: https://pairedomicsdata.bioinformatics.nl/projects/0ff7a302-49af-4130-a440-59e284d4d365.4, https://pairedomicsdata.bioinformatics.nl/projects/297c364c-b154-4edd-a7d5-68decf9effa2.4, and https://pairedomicsdata.bioinformatics.nl/projects/4b29ddc3-26d0-40d7-80c5-44fb6631dbf9.4. The RefSeq and GenBank accessions for the strains from the Cyanobacteria and Nocardia dataset are available in the following PoDP accessions: https://pairedomicsdata.bioinformatics.nl/projects/84b56cd3-218b-4e57-906f-a9003615ed07.2, and https://pairedomicsdata.bioinformatics.nl/projects/a4837d37-1df1-4153-b365-c06841574235.3.

## Abbreviations

MS/MS: Mass fragmentation spectra
BGC: Biosynthetic gene cluster
NP: Natural product
PoDP: Paired Omics Data Platform
GCF: Gene cluster family
MF: Molecular family
NPClassScore: NPLinker Class-based matching Score for linking BGCs and MS/MS-spectra
NRP: Non-ribosomal peptide
PK: Polyketide
OrgHetCyc: Organoheterocyclic compounds
