# Supplementary material for: Enhanced correlation-based linking of biosynthetic gene clusters to their metabolic products through chemical class matching
Source: Microbiome. 2023 Jan 23;11:13. doi: 10.1186/s40168-022-01444-3 (PMC9869629; doi:10.1186/s40168-022-01444-3)
Supplement: Supplementary file 2 — Additional file 1: Figure S1. MIBiG classes matched to ClassyFire superclasses. Figure S2. antiSMASH-predicted classes matched to NPClassifier superclasses, where matches with counts above five are shown. Figure S3. antiSMASH-predicted classes matched to ClassyFire classes, where matches with counts above five are shown. Figure S4. Number of links with MS/MS spectra per GCF for all the GCFs in the Streptomyces/Salinispora dataset after using standardised Metcalf scoring in combination with NPClassScore filtering at varying cut-offs for the NPClassScore. The standardised Metcalf score cut-off was 2.5. Figure S5. Number of links with MS/MS spectra per GCF for all the GCFs in the Cyanobacteria dataset after using standardised Metcalf scoring in combination with NPClassScore filtering at varying cut-offs for the NPClassScore. The standardised Metcalf score cut-off was 2.5. Figure S6. Number of links with MS/MS spectra per GCF for all the GCFs in the Nocardia dataset after using standardised Metcalf scoring in combination with NPClassScore filtering at varying cut-offs for the NPClassScore. The standardised Metcalf score cut-off was 2.5. Figure S7. Histograms showing the number of candidate MS/MS spectrum links per GCF in the (a) Cyanobacteria dataset and (b) Nocardia dataset after co-occurrence scoring (standardised Metcalf), and after NPClassScore filtering with a cut-off of 0.25. The bin sizes are 5 in (a) and 25 in (b). The results highlight how NPClassScore narrows down the number of candidate links for most GCFs. Figure S8. Number of retained validated MS/MS spectrum links versus the percentage of filtered out candidate links per GCFs with different NPClassScore cut-offs. The percentage of filtered out candidate links is an average over the three datasets. Table S1. Scoring table from NPClassScore showing the scores from MIBiG classes to NPClassifier pathways. Table S2. Translation of BiG-SCAPE to MIBiG classes. Table S3. Translation of antiSMASH classes from all o [file 40168_2022_1444_MOESM1_ESM.docx]

Supplemental Information for:

Enhanced correlation-based linking of biosynthetic gene clusters to their metabolic products through chemical class matching

Joris J. R. Louwen^1^, Marnix H. Medema^1^, Justin J. J. van der Hooft^1,2*^

1. Bioinformatics Group, Wageningen University, 6708PB Wageningen, The Netherlands

2. Department of Biochemistry, University of Johannesburg, Johannesburg 2006, South Africa

* Corresponding author - justin.vanderhooft@wur.nl

**Supplementary Figures**


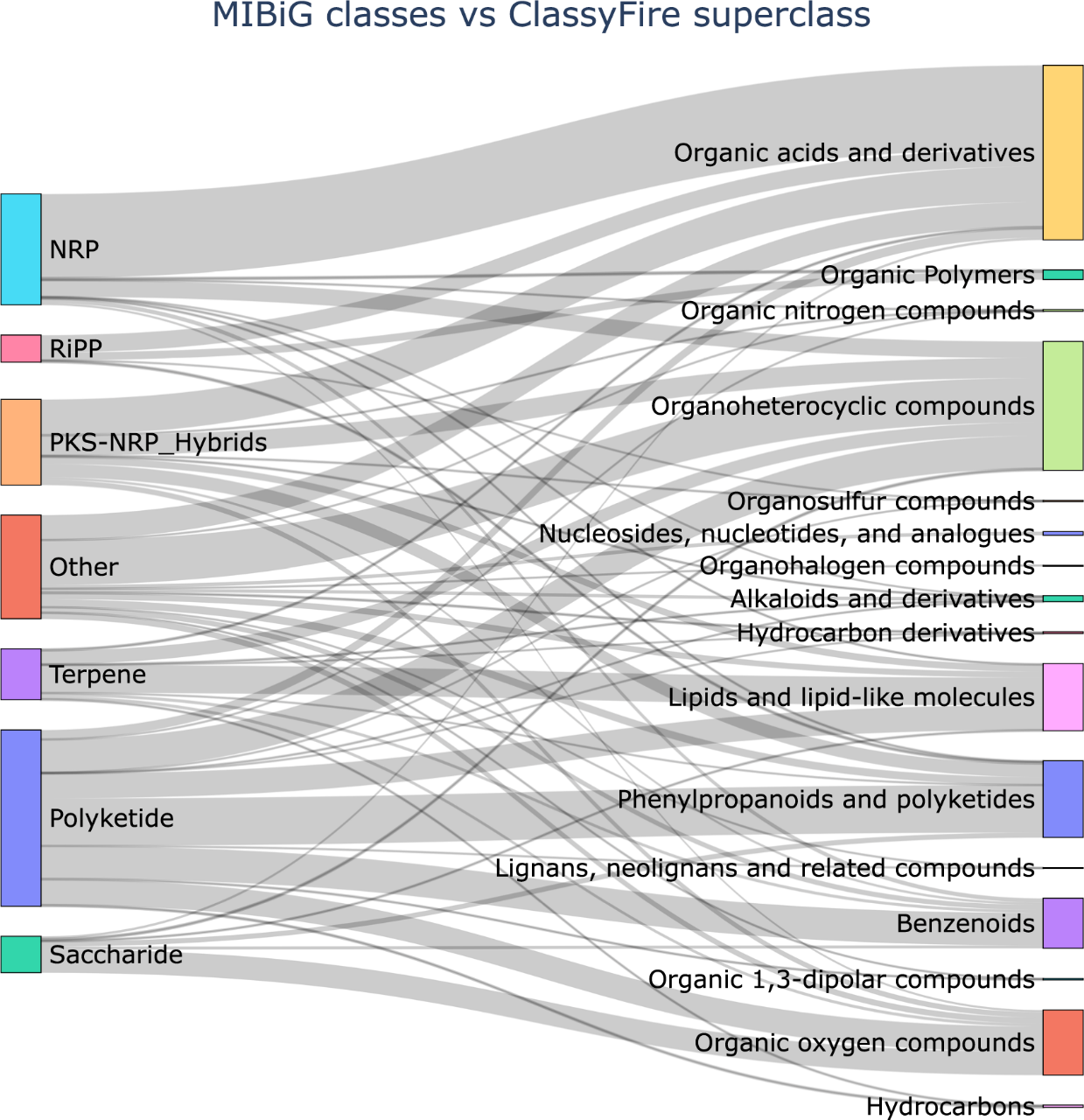


**Figure S1.** MIBiG classes matched to ClassyFire superclasses.


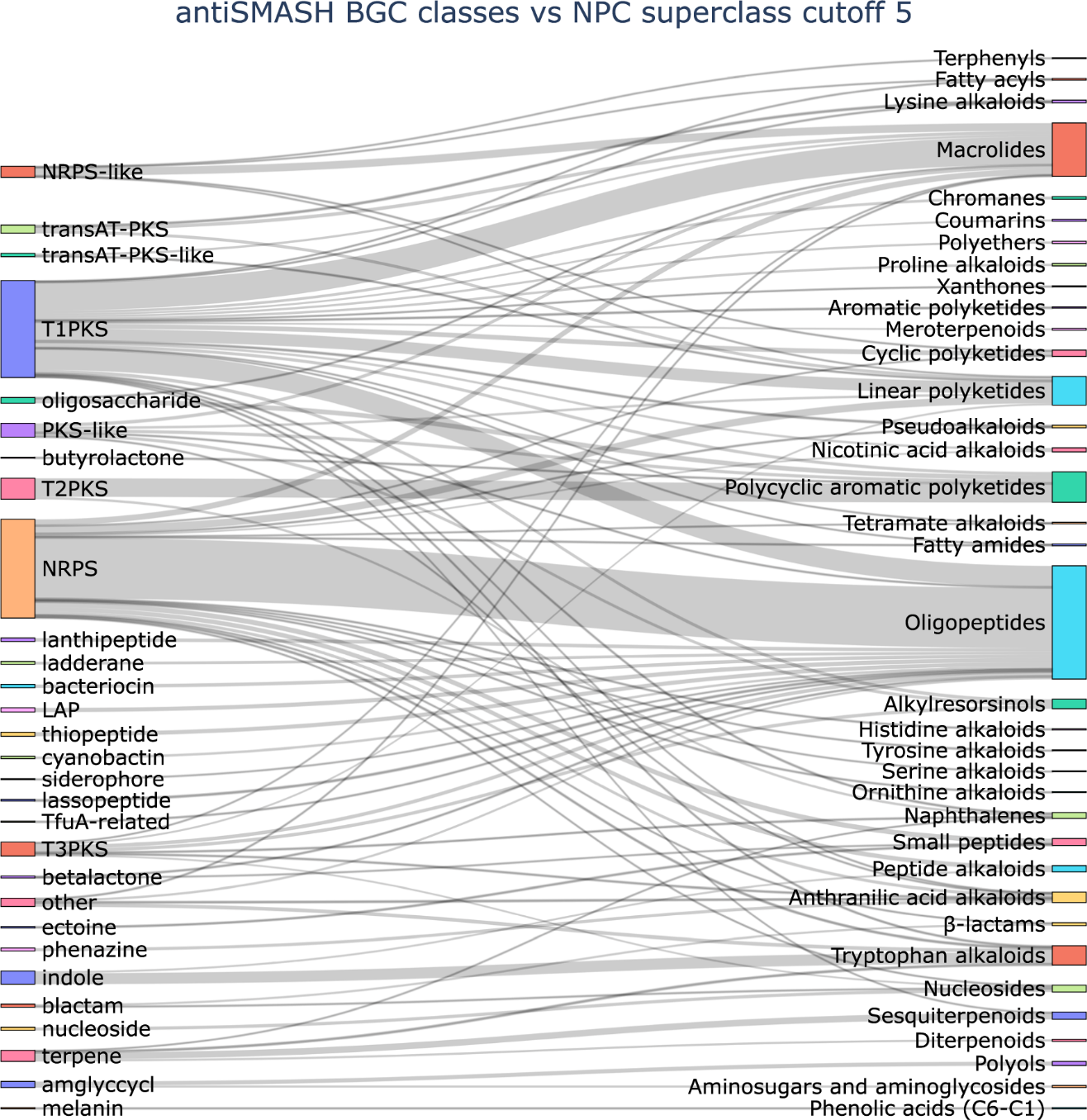


**Figure S2.** antiSMASH-predicted classes matched to NPClassifier superclasses, where matches with counts above five are shown.


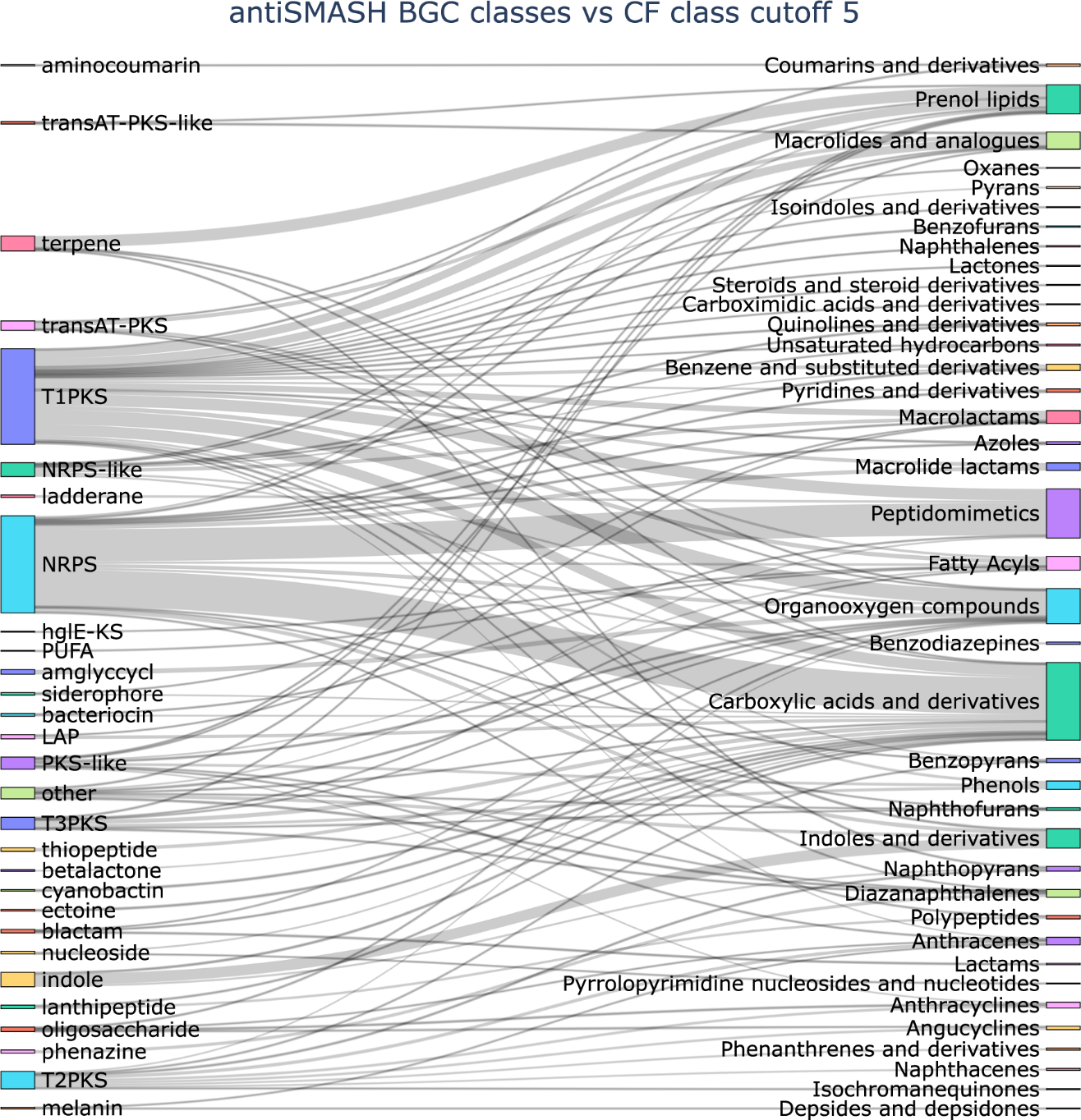


**Figure S3.** antiSMASH predicted classes matched to ClassyFire classes, where matches with counts above five are shown.


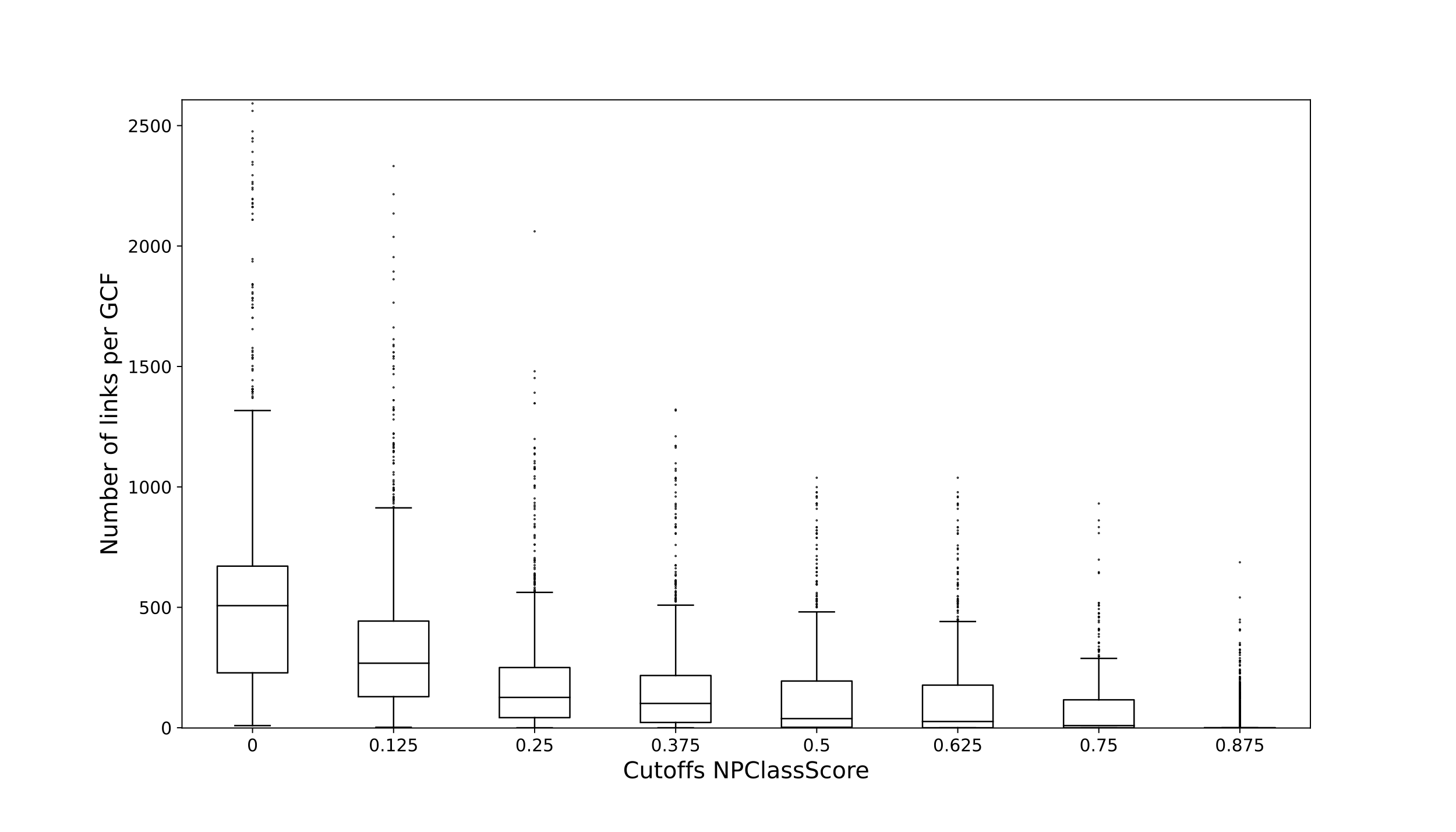


**Figure S4.** Number of links with MS/MS spectra per GCF for all the GCFs in the Streptomyces/Salinispora dataset after using standardised Metcalf scoring in combination with NPClassScore filtering at varying cut-offs for the NPClassScore. The standardised Metcalf score cut-off was 2.5.


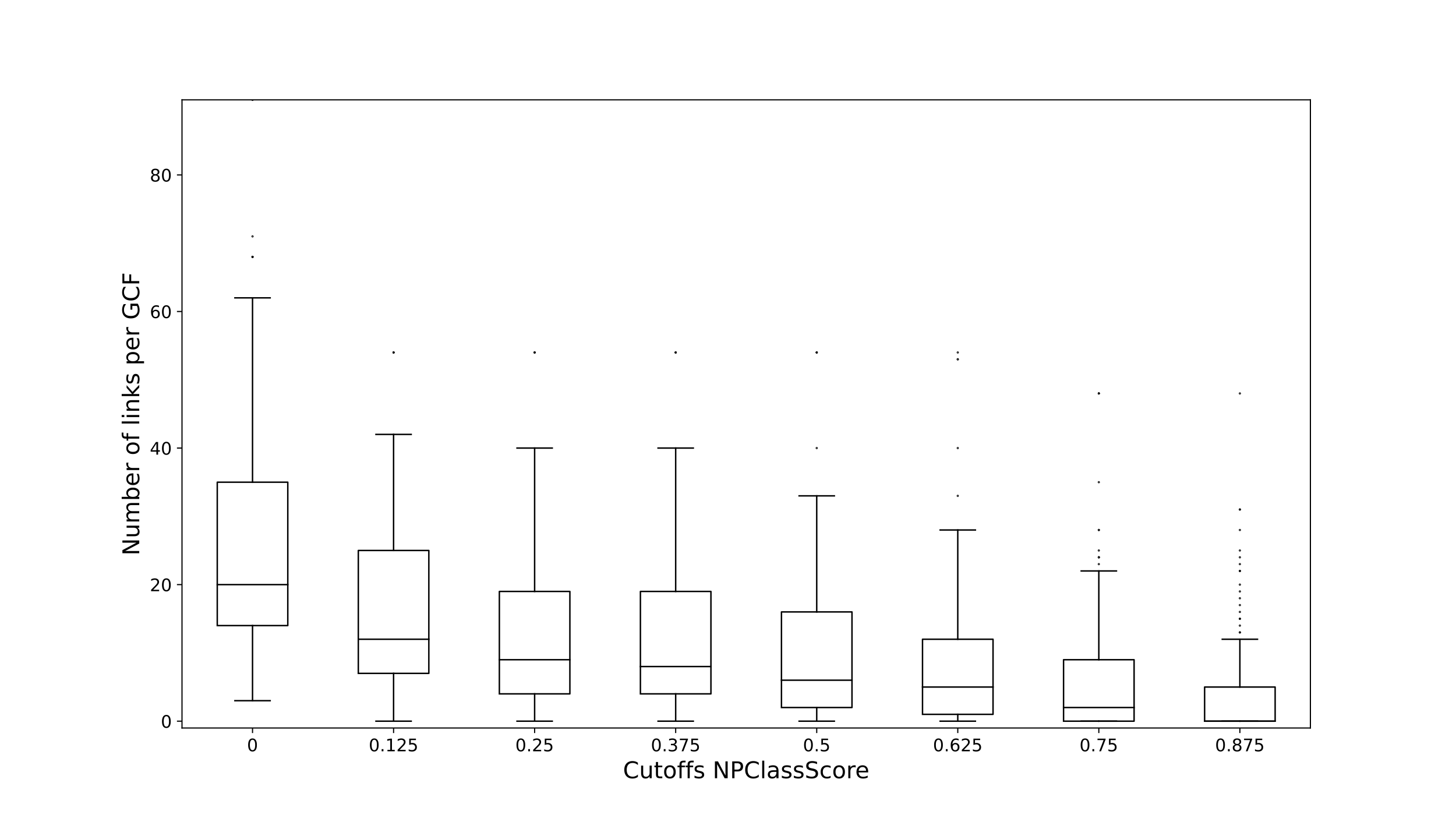


**Figure S5.** Number of links with MS/MS spectra per GCF for all the GCFs in the Cyanobacteria dataset after using standardised Metcalf scoring in combination with NPClassScore filtering at varying cut-offs for the NPClassScore. The standardised Metcalf score cut-off was 2.5.


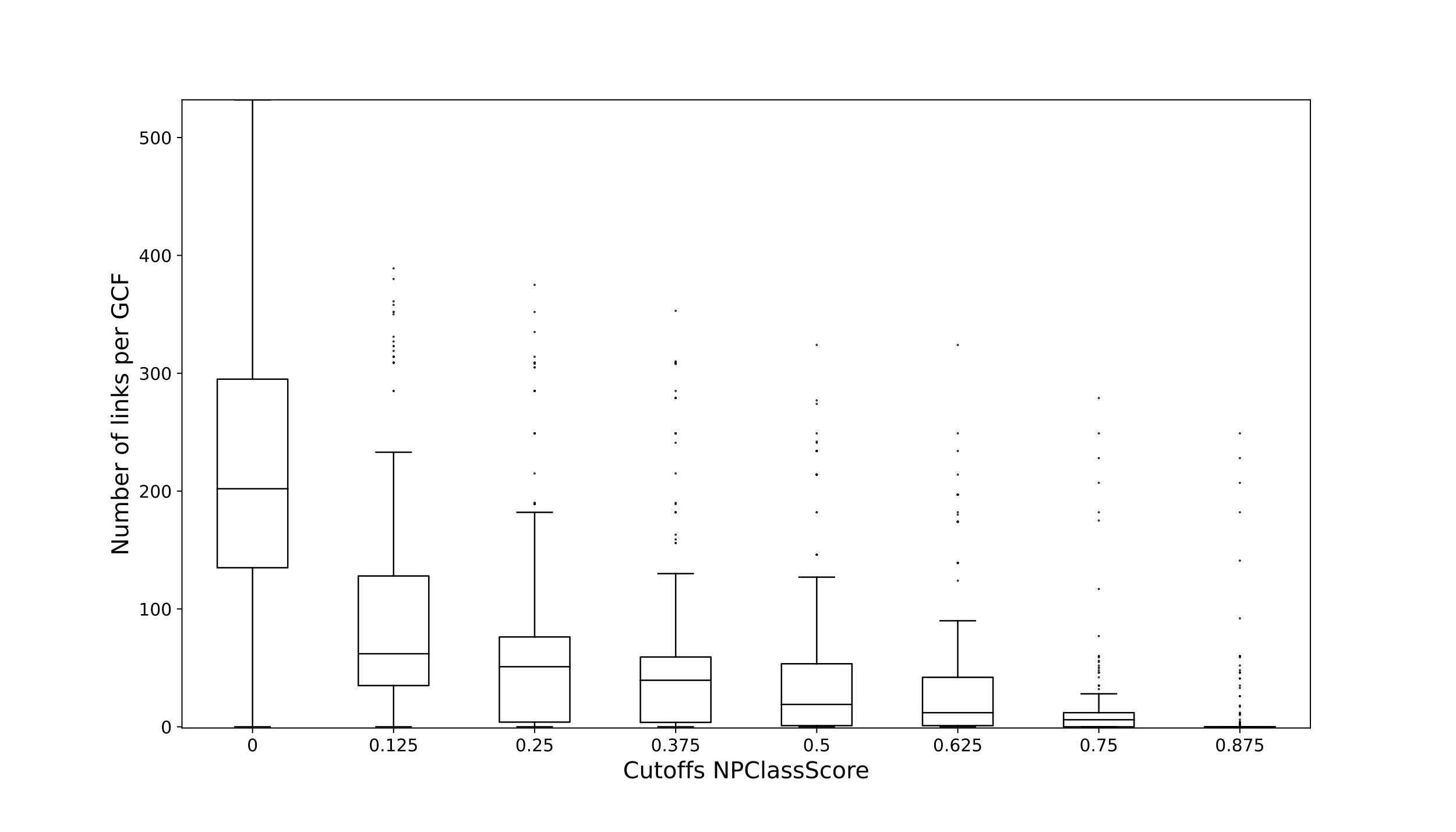


**Figure S6.** Number of links with MS/MS spectra per GCF for all the GCFs in the Nocardia dataset after using standardised Metcalf scoring in combination with NPClassScore filtering at varying cut-offs for the NPClassScore. The standardised Metcalf score cut-off was 2.5.


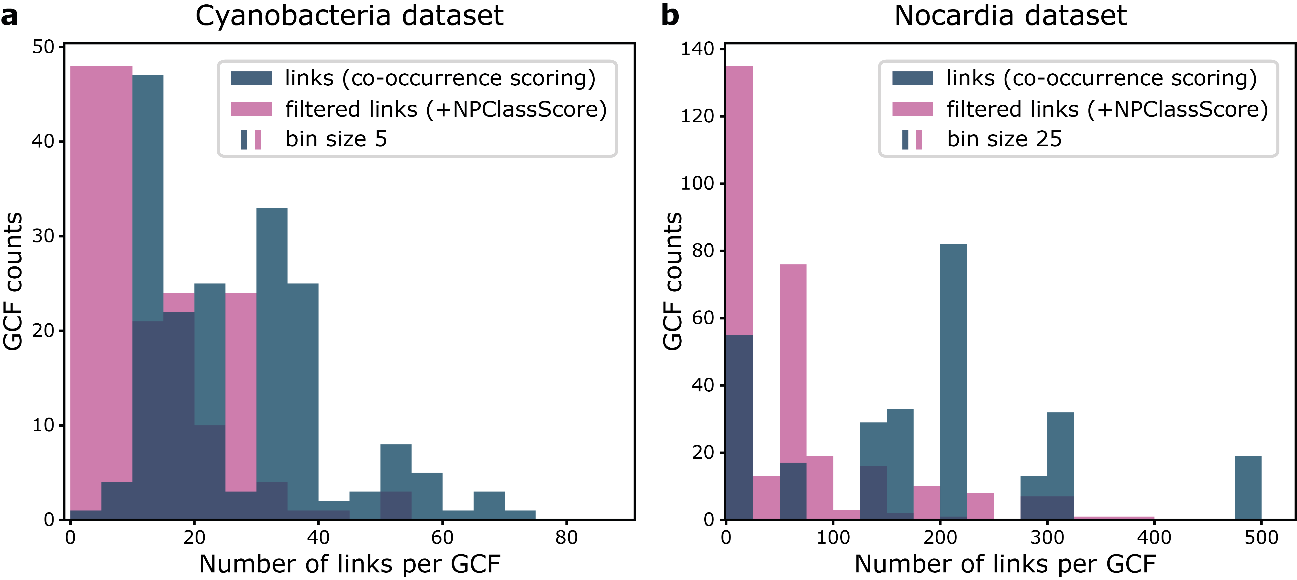


**Figure S7.** Histograms showing the number of candidate MS/MS spectrum links per GCF in the (a) Cyanobacteria dataset and (b) Nocardia dataset after co-occurrence scoring (standardised Metcalf), and after NPClassScore filtering with a cut-off of 0.25. The bin sizes are 5 in (a) and 25 in (b). The results highlight how NPClassScore narrows down the number of candidate links for most GCFs.


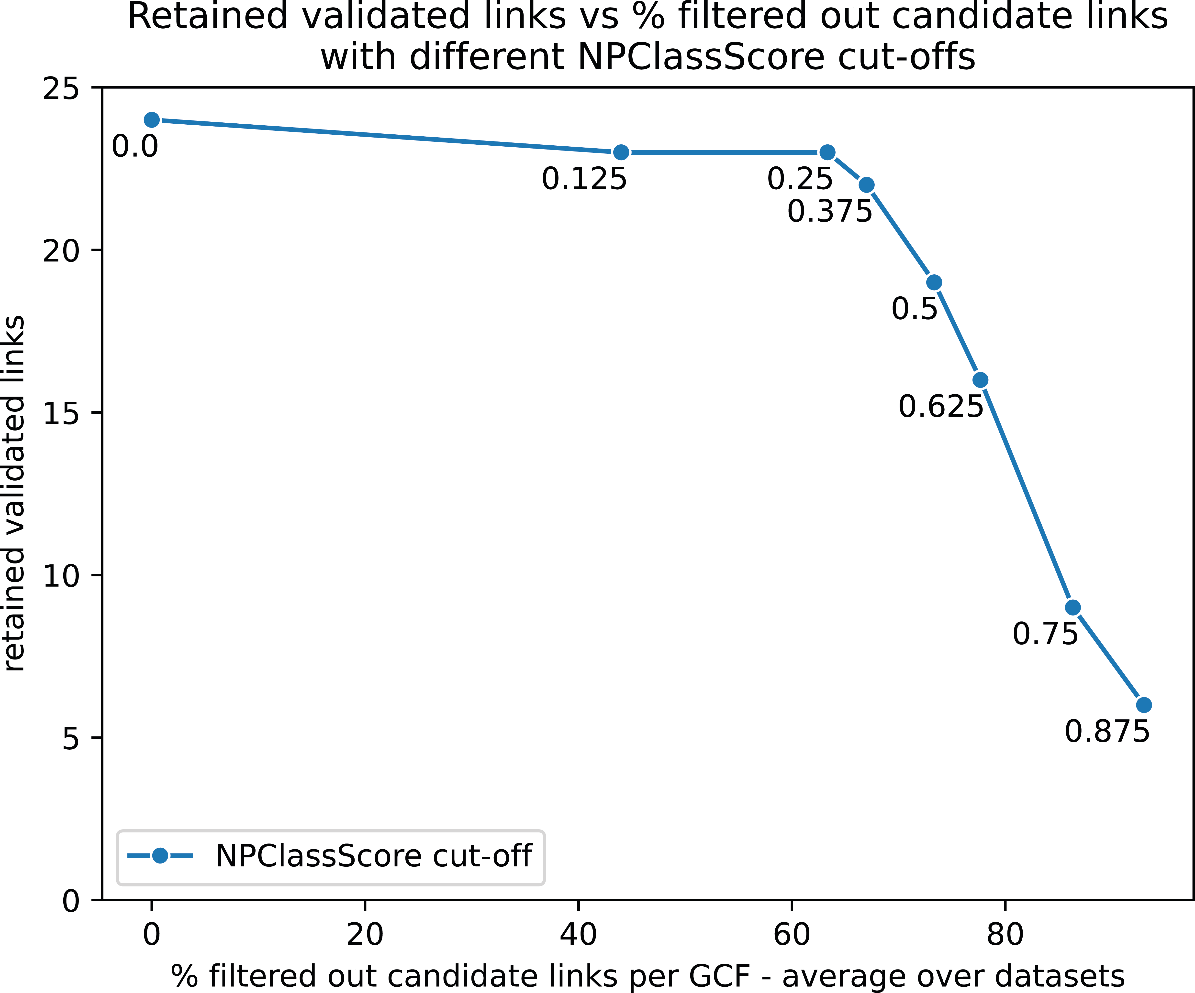


**Figure S8.** Number of retained validated MS/MS spectrum links versus the percentage of filtered out candidate links per GCFs with different NPClassScore cut-offs. The percentage of filtered out candidate links is an average over the three datasets.

**Supplementary Tables**

**Table S1.** Scoring table from NPClassScore showing the scores from MIBiG classes to NPClassifier pathways.

|  | **Polyketide** | **Other** | **Saccharide** | **Terpene** | **PKS-NRP_Hybrids** | **NRP** | **RiPP** |
| --- | --- | --- | --- | --- | --- | --- | --- |
| **Polyketides** | 0.759 | 0.088 | 0.457 | 0.091 | 0.400 | 0.208 | 0.034 |
| **Shikimates and Phenylpropanoids** | 0.028 | 0.051 | 0.021 | 0.005 | 0.014 | 0.016 | 0.000 |
| **Alkaloids** | 0.100 | 0.471 | 0.000 | 0.236 | 0.131 | 0.179 | 0.017 |
| **Amino acids and Peptides** | 0.033 | 0.220 | 0.064 | 0.010 | 0.422 | 0.579 | 0.950 |
| **Terpenoids** | 0.048 | 0.012 | 0.029 | 0.654 | 0.005 | 0.000 | 0.000 |
| **Fatty acids** | 0.019 | 0.046 | 0.000 | 0.000 | 0.027 | 0.004 | 0.000 |
| **Carbohydrates** | 0.012 | 0.112 | 0.429 | 0.005 | 0.002 | 0.014 | 0.000 |

**Table S2.** Translation of BiG-SCAPE to MIBiG classes.

| **BiG-SCAPE class** | **MIBiG class** |
| --- | --- |
| PKSI | Polyketide |
| PKSother | Polyketide |
| NRPS | NRP |
| RiPPs | RiPP |
| Saccharides | Saccharide |
| Others | Other |
| Terpene | Terpene |
| PKS-NRP_Hybrids | PKS-NRP_Hybrids |

**Table S3.** Translation of antiSMASH classes from all other versions to match antiSMASH v5 classes.

| **original antiSMASH class** | **antiSMASH v5 class** |
| --- | --- |
| NAGGN | other |
| NAPAA | other |
| RRE-containing | bacteriocin |
| RiPP-like | bacteriocin |
| cf_fatty_acid | fatty_acid |
| cf_putative | other |
| cf_saccharide | saccharide |
| guanidinotides | fused |
| lanthipeptide-class-i | lanthipeptide |
| lanthipeptide-class-ii | lanthipeptide |
| lanthipeptide-class-iii | lanthipeptide |
| lanthipeptide-class-iv | lanthipeptide |
| lanthipeptide-class-v | lanthipeptide |
| lantipeptide | lanthipeptide |
| linaridin | lanthipeptide |
| lipolanthine | lanthipeptide |
| nrps | NRPS |
| otherks | hglE-KS |
| prodigiosin | other |
| pyrrolidine | other |
| ranthipeptide | bacteriocin |
| redox-cofactor | other |
| t1pks | T1PKS |
| t2pks | T2PKS |
| t3pks | T3PKS |
| thioamide-NRP | other |
| thioamitides | bacteriocin |
| transatpks | transAT-PKS |

**Table S4.** Information about the contents of the three datasets. We included the number of spectra in our versions of the datasets separately as some spectra present in the molecular networks occurred in, for example, control samples, or samples we did not use.

| # | **Streptomyces/Salinispora** | **Cyanobacteria** | **Nocardia** |
| --- | --- | --- | --- |
| strains | 154 | 24 | 11 |
| BGCs | 5869 | 301 | 367 |
| GCFs | 1581 | 184 | 300 |
| spectra | 13667 | 729 | 11464 |
| MFs | 8346 | 341 | 10458 |
| spectra in our version of the data | 13381 | 707 | 3080 |
| spectra without structure-based class prediction | 1145 | 34 | 809 |

**Table S5.** The average number of MS/MS spectrum links per GCF for each dataset after NPClassScore filtering with different cut-offs, along with the percentual decrease in the number of links. In this case, the MS/MS spectra without structure-based predictions are automatically excluded.

| **NPClassScore cutoff** | **Streptomyces/Salinispora** | | **Cyanobacteria** | | **Nocardia** | |
| --- | --- | --- | --- | --- | --- | --- |
|  | Number of links | Change | Number of links | Change | Number of links | Change |
| 0 | 549.85 | 0 | 26.98 | 0 | 206.33 | 0 |
| 0.125 | 332.96 | -39 | 15.81 | -41 | 98.27 | -52 |
| 0.25 | 177.00 | -68 | 12.61 | -53 | 64.35 | -69 |
| 0.375 | 149.46 | -73 | 11.78 | -56 | 58.24 | -72 |
| 0.5 | 115.89 | -79 | 9.88 | -63 | 45.85 | -78 |
| 0.625 | 105.10 | -81 | 8.42 | -69 | 35.17 | -83 |
| 0.75 | 64.19 | -88 | 6.20 | -77 | 13.03 | -94 |
| 0.875 | 23.13 | -96 | 3.82 | -86 | 6.31 | -97 |

**Table S6.** Counts for the number of spectra with certain ClassyFire superclasses as predicted by MolNetEnhancer (MNE), MNE for spectra below 850 Da, and CANOPUS. Counts are coloured from white to red, white being the lowest count and red being the highest count for each column.

| **CF superclasses** | **MNE predicted spectra** | **MNE predicted spectra <850Da** | **CANOPUS predicted spectra** |
| --- | --- | --- | --- |
| Alkaloids and derivatives | 173 | 150 | 9 |
| Benzenoids | 281 | 263 | 522 |
| Hydrocarbon derivatives | 17 | 14 | 13 |
| Hydrocarbons | 0 | 0 | 1 |
| Lignans, neolignans and related compounds | 61 | 60 | 3 |
| Lipids and lipid-like molecules | 4597 | 3603 | 1647 |
| Nucleosides, nucleotides, and analogues | 18 | 18 | 136 |
| Organic 1,3-dipolar compounds | 0 | 0 | 30 |
| Organic Polymers | 105 | 10 | 2 |
| Organic acids and derivatives | 2437 | 1865 | 4057 |
| Organic nitrogen compounds | 79 | 78 | 280 |
| Organic oxygen compounds | 231 | 209 | 882 |
| Organohalogen compounds | 16 | 16 | 0 |
| Organoheterocyclic compounds | 1013 | 981 | 731 |
| Organophosphorus compounds | 0 | 0 | 2 |
| Organosulfur compounds | 1 | 1 | 29 |
| Phenylpropanoids and polyketides | 1528 | 1373 | 202 |
| Total | 10557 | 8641 | 8546 |

**Table S7.** Counts of the ClassyFire superclass predictions for the 6,606 spectra that could be predicted by both MolNetEnhancer (MNE), and CANOPUS, showing the MNE predictions and CANOPUS predictions. Counts are coloured from white to red, white being the lowest count and red being the highest count for each column.

| **CF superclasses** | **MNE predictions** | **CANOPUS predictions** |
| --- | --- | --- |
| Alkaloids and derivatives | 119 | 7 |
| Benzenoids | 203 | 338 |
| Hydrocarbon derivatives | 8 | 13 |
| Hydrocarbons | 0 | 1 |
| Lignans, neolignans and related compounds | 33 | 3 |
| Lipids and lipid-like molecules | 2686 | 1301 |
| Nucleosides, nucleotides, and analogues | 16 | 81 |
| Organic 1,3-dipolar compounds | 0 | 16 |
| Organic Polymers | 10 | 1 |
| Organic acids and derivatives | 1553 | 3229 |
| Organic nitrogen compounds | 72 | 243 |
| Organic oxygen compounds | 165 | 700 |
| Organohalogen compounds | 16 | 0 |
| Organoheterocyclic compounds | 773 | 534 |
| Organophosphorus compounds | 0 | 2 |
| Organosulfur compounds | 1 | 24 |
| Phenylpropanoids and polyketides | 951 | 113 |
| Total | 6606 | 6606 |

**Table S8.** The average number of candidate links per GCF for each dataset after NPClassScore filtering with different cut-offs, along with the percentual decrease in the number of links. In this case, the MS/MS spectra without structure-based predictions are automatically included.

| **NPClassScore cutoff** | **Streptomyces/Salinispora** | | **Cyanobacteria** | | **Nocardia** | |
| --- | --- | --- | --- | --- | --- | --- |
|  | Number of links | Change | Number of links | Change | Number of links | Change |
| 0 | 549.85 | 0 | 26.98 | 0 | 206.33 | 0 |
| 0.125 | 372.64 | -32 | 17.32 | -36 | 158.98 | -23 |
| 0.25 | 216.68 | -61 | 14.12 | -48 | 125.05 | -39 |
| 0.375 | 189.14 | -66 | 13.29 | -51 | 118.95 | -42 |
| 0.5 | 155.57 | -72 | 11.39 | -58 | 106.56 | -48 |
| 0.625 | 144.78 | -74 | 9.93 | -63 | 95.88 | -54 |
| 0.75 | 103.87 | -81 | 7.71 | -71 | 73.73 | -64 |
| 0.875 | 62.81 | -89 | 5.33 | -80 | 67.01 | -68 |
